# Qingwenzhike Prescription Alleviates Acute Lung Injury Induced by LPS *via* Inhibiting TLR4/NF-kB Pathway and NLRP3 Inflammasome Activation

**DOI:** 10.3389/fphar.2021.790072

**Published:** 2021-12-23

**Authors:** Cai Zhang, Xinran Wang, Chunguo Wang, Cheng He, Quantao Ma, Jialin Li, Weiling Wang, Yan-Tong Xu, Ting Wang

**Affiliations:** ^1^ Beijing Research Institute of Chinese Medicine, Beijing University of Chinese Medicine, Beijing, China; ^2^ NMPA Key Laboratory for Research and Evaluation of Traditional Chinese Medicine, Beijing University of Chinese Medicine, Beijing, China; ^3^ School of Chinese Materia Medica, Beijing University of Chinese Medicine, Beijing, China

**Keywords:** Acute Lung Injury (ALI), Qingwenzhike (QWZK) prescription, inflammation cytokines, TLR4/NF-κB signaling pathway, inflammasome, NLRP3

## Abstract

**Background:** Acute lung injury (ALI) is characterized by dysfunction of the alveolar epithelial membrane caused by acute inflammation and tissue injury. Qingwenzhike (QWZK) prescription has been demonstrated to be effective against respiratory viral infections in clinical practices, including coronavirus disease 2019 (COVID-19) infection. So far, the chemical compositions, protective effects on ALI, and possible anti-inflammatory mechanisms remain unknown.

**Methods:** In this study, the compositions of QWZK were determined *via* the linear ion trap/electrostatic field orbital trap tandem high-resolution mass spectrometry (UHPLC-LTQ-Orbitrap MS). To test the protective effects of QWZK on ALI, an ALI model induced by lipopolysaccharide (LPS) in rats was used. The effects of QWZK on the LPS-induced ALI were evaluated by pathological changes and the number and classification of white blood cell (WBC) in bronchoalveolar lavage fluid (BALF). To investigate the possible underlying mechanisms, the contents of interleukin-6 (IL-6), tumor necrosis factor-α (TNF-α), monocyte chemoattractant protein (MCP-1), interleukin-1β (IL-1β), interleukin-18 (IL-18), and immunoregulatory-related factors interferon-γ (IFN-γ) were detected by ELISA. Furthermore, the expression of Toll-like receptor 4 (TLR4), p-IKKα/β, IKKα, IKKβ, p-IκBα, IκBα, p-NF-κB, nuclear factor-κB (NF-κB), NOD-like receptor family pyrin domain containing 3 (NLRP3), cleaved caspase-1, pro-caspase-1, apoptosis-associated speck-like protein containing CARD (ASC), and β-actin were tested by Western blot.

**Results:** A total of 99 compounds were identified in QWZK, including 33 flavonoids, 23 phenolic acids, 3 alkaloids, 3 coumarins, 20 triterpenoids, 5 anthraquinones, and 12 others. ALI rats induced by LPS exhibited significant increase in neutrophile, significant decrease in lymphocyte, and evidently thicker alveolar wall than control animals. QWZK reversed the changes in WBC count and alveolar wall to normal level on the model of ALI induced by LPS. ELISA results revealed that QWZK significantly reduced the overexpression of proinflammatory factors IL-6, TNF-α, MCP-1, IL-1β, IL-18, and IFN-γ induced by LPS. Western blot results demonstrated that QWZK significantly downregulated the overexpression of TLR4, p-IKKα/β, p-IκBα, p-NF-κB, NLRP3, cleaved caspase-1, and ASC induced by LPS, which suggested that QWZK inhibited TLR4/NF-κB signaling pathway and NLRP3 inflammasomes.

**Conclusions:** The chemical compositions of QWZK were first identified. It was demonstrated that QWZK showed protective effects on ALI induced by LPS. The possible underlying mechanisms of QWZK on ALI induced by LPS was *via* inhibiting TLR4/NF-kB signaling pathway and NLRP3 inflammasome activation. This work suggested that QWZK is a potential therapeutic candidate for the treatments of ALI and pulmonary inflammation.

## Introduction

The coronavirus disease 2019 (COVID-19) pandemic causes tremendous catastrophe worldwide. During the development of COVID-19 infection. Acute lung injury (ALI) is a critical step and causes high mortality (Leist et al., 2020; Lin et al., 2021). In ALI, the lungs show widespread destruction of the capillary endothelium, damages in alveolar capillary barrier, lung inflammatory cell infiltration, diffuse alveolar, and pulmonary interstitial edema, which lead to respiratory distress, progressive hypoxemia, and acute respiratory distress syndrome (ARDS) (Gupta et al., 2020; England et al., 2021). ALI/ARDS induced by COVID-19 overproduces early response proinflammatory cytokines TNF-α, interleukin (IL)-6, and IL-1β, which results in cytokine storm, and then leads to vascular hyperpermeability, multiorgan failure, high cytokine concentrations unabated over time, and eventually death (England et al., 2021). Therefore, it is critical to develop protective treatments against ALI.

The most common risk factors for ALI are severe infections (e.g., sepsis/septic shock) and pneumonia induced by various microbial pathogens, such as bacteria, viruses, fungi, *rickettsia*, and parasites. Due to the limitation in availability of high levels of bio-safety labs for antiviral studies, lipopolysaccharide (LPS) has been used extensively in studies on inflammatory diseases. LPS is a major microbial mediator in Gram-negative bacterial infection (Ratajczak and Kucia, 2020), and Toll-like receptors (TLRs) are the transmembrane transduction receptors for LPS signaling from extracellular to intracellular space. LPS directly binds to TLR4 to activate the NF-κB signaling pathway, which leads to the synthesis and release of various inflammatory mediators, and finally initiates and amplifies the inflammatory responses (N. Li et al., 2020; Li et al., 2017). Meanwhile, inhibition on TLR4/NF-κB pathway attenuated the injury and inflammation of the lung tissues in ALI (Ciesielska et al., 2021; Rosadini & Kagan, 2017; N. ; Yang et al., 2016). Thus, TLR4/NF-κB pathway plays an important role in LPS infections. Besides TLR4/NF-κB pathway, it has been recently unveiled that activation of NLRP3 inflammasome is another critical mechanism during ALI. NLRP3 can regulate the manufacture of IL-1β and IL-18. Through binding to the adaptor ASC, NLRP3 induces pro-Caspase-1 recruitment, auto-activation and pro-IL-1β and pro-IL-18 shear processing, and responds to diverse incentive, including ATP, bacterial toxins, bacteria and viruses (Afonina et al., 2017). It has also been identified as an important target for pneumonia, asthma, sepsis, or chronic obstructive pulmonary disease (COPD) (Scambler et al., 2018; Theofani et al., 2019; Pearce et al., 2021). So TLR4, NF-κB, NLRP3 inflammasome have been considered as promising pharmacological targets for inflammatory diseases, including ALI and pneumonia (Du et al., 2019; Wu et al., 2020; Yao et al., 2017).

Current clinical therapeutic drug for ALI is corticosteroids (CS). On the one hand, it exerts a wide spectrum of bioactivities including anti-inflammatory, antioxidant, pulmonary vasodilator, and antiedematous effects; on the other hand, side effects of CS are evident, such as immunosuppression, osteoporosis, and peptic ulcers (Vandewalle et al., 2018). Although several cytokine-targeted therapies, such as tocilizumab and anakinra, are currently being used to treat the observed cytokine storm associated with COVID-19 (Kim et al., 2021), they were only used to treat critical phase patients. Thus, there are still tremendous unmet needs for treatments of COVID-19.

Traditional Chinese medicine (TCM) has a long history in clinical practices in China. During the COVID-19 pandemic, TCM has been widely used in China and has been demonstrated to show convincing effects. Qingwenzhike (QWZK) is a TCM preparation derived from recombination of ancient Chinese classical prescriptions, including Maxingshigan decoction (Cheng et al., 2019), Sheganmahuang prescription, and Shengjiang powder. It has been applied to treat acute phase of COVID-19 infection and was demonstrated to be effective in mild type of COVID-19 patients in Wuhan. Moreover, QWZK was approved as a hospital preparation by the Beijing government used in COVID-19 treatments in Beijing region during the outbreak period. More than that, an international cooperation program on clinical trials of QWZK for treatments on COVID-19 are being untaken in South Africa. So far, the chemical compositions, protective effects against ALI, and possible action mechanisms of QWZK prescription remain unknown. In the present study, the chemical compositions of QWZK were determined. The protective effects of QWZK on ALI was evaluated on a rat model stimulated by LPS. The possible mechanisms of QWZK were supposed by inhibiting TLR4/NF-κB pathway and NLRP3 inflammasome in cytokine expression.

## Materials and Methods

### Chemicals and Reagents

Ephedrae Herba (No. 20200103), Gypsum Fibrosum (No. 20200327), Rhei Radix Et Rhizoma (No. 20200107), Belamcandae Rhizoma (No. 20200412), Asteris Radix Et Rhizoma (No. 20200310), Farfarae Flos (No. 20200411), Citri Reticulatae Pericarpium (No. 20200511), Pinelliae Rhizoma Praeparatum Cum Zingibere Et Alumin (No. 20200717), Poria (No. 20200904), Armeniacae Semen Amarum (No. 20200414), Cicadae Periostracum (No. 20200813), Fritillariae Thunbergii Bulbus (No. 20200907), Taraxaci Herba (No. 20200528), and Platycodonis Radix (No. 20200816) were provided by Beijing Bencaofangyuan Pharmaceutical Co., Ltd. (Beijing, China). The standards including alanine, caffeic acid, quercetin, β-sitosterol, chrysophanol, amygdalin, and hesperidin were purchased from National Institutes for Food and Drug Control. Lipopolysaccharides (LPS, from *Escherichia coli* O55:B5, abs47014848, Absin, Shanghai, China) and dexamethasone (D4902, Sigma-Aldrich, St. Louis, MO, United States) were purchased from Absin and Sigma-Aldrich.

### Preparation of QWZK

QWZK comprises 14 herbs: Ephedrae Herba, Gypsum Fibrosum, Rhei Radix Et Rhizoma, Belamcandae Rhizoma, Asteris Radix Et Rhizoma, Farfarae Flos, Citri Reticulatae Pericarpium, Pinelliae Rhizoma Praeparatum Cum Zingibere Et Alumin, Poria, 6.30 % Armeniacae Semen Amarum, Cicadae Periostracum, Fritillariae Thunbergii Bulbus, Taraxaci Herba, and Platycodonis Radix. The QWZK was acquired as described above. The specimens (No. 20211009) were deposited in the Beijing Research Institute of Chinese Medicine, Beijing University of Chinese Medicine.

The preparation methods of the QWZK powder were as follows: the crude drugs of QWZK accurately weighed 1.43 kg. These drugs were soaked in 14.30 L (10 times, w/v) pure water for 30 min and was then boiled for 2 h. Subsequently, they were boiled in 11.40 L (8 times, w/v) pure water for 1 h twice.

The extracts were filtered through three-layer gauze, then combined and concentrated to 66.50 ml in a rotary evaporator at 75°C. The concentrate was vacuum dried, and 0.446 kg dry powder was obtained. The extract rate (%) = extract dry powder/total quality of crude drugs. Therefore, the extract rate of QWZK powder was 31.20%. The powder was used in the follow-up experiments.

According to the body surface area (BSA) scaling for converting the dose of a test drug from human clinical trials to animal species (Mahmood, 2007; Blanchard & Smoliga, 2015), the test doses of QWZK in animals were 3, 6, and 12 g/kg/day and that of an adult human was 71.5 g/day of crude drugs. The body weight of an adult human is 70 kg, and the convert coefficient is 6. Based on the extract rate, the text dose of QWZK powder were 0.94, 1.87, and 3.74 g/ kg/ day in animals.

### Quantitative Analysis of QWZK

Liquid chromatography was performed using a Dionex Utimate 3000 UHPLC Plus Focused Ultra High-Performance Liquid Chromatography System (Thermo Scientific, Santa Clara, CA, United States). Chromatographic separation was achieved through a ACQUITY UPLC C18 column (2.1 mm × 100 mm, 1.7 mm particles) at a flowrate of 0.3 ml/ min, defended by a high-pressure column prefilter (2 mm) (Shimadzu, Kyoto, Japan) at 35°C. Mass spectrometric detection was performed with an LTQ-Oribitrap XL linear ion trap tandem electrostatic field orbital trap mass spectrometer (Thermo Scientific, Santa Clara, CA, United States) in positive and negative ion modes, which was equipped with an electrospray ion source in MRM modes.

QWZK powder was accurately weighed 1.00 g and added into 10.00 ml methanol, ultrasonically treated for 45 min using an ultrasonic cleaning instrument (KQ-500DB CNC, Kunshan Ultrasonic Instrument Co., Ltd., Kunshan, Jiangsu, China), and the solution was filtrated through 0.22 μm microporous membrane. The standards such as alanine, caffeic acid, quercetin, β-sitosterol, chrysophanol, amygdalin, and hesperidin were weighed in precision, dissolved in methanol with a standard solution of 1 mg/ml, and filtered through 0.22 μm microporous membrane. Samples or strands were separated on an ACQUITY UPLC C18 column (2.1 mm × 100 mm, 1.7 mm) at 35°C. The mobile phase consisted of 0.1% formic acid aqueous solution (A) and acetonitrile solution (B). The gradient elution conditions were as follows: 0–6 min (90–60% A), 6–9 min (60–40% A), 9–42 min (40–20% A), and 42–60 min (20–90% A). The flowrate was 0.3 ml/min, and the injection volume was 3.0 μl.

Electrospray ionization mass spectrometry (ESI-MSP) analyses were performed on an LTQ-Oribitrap XL linear ion trap tandem electrostatic field orbital trap mass spectrometer (Thermo Scientific, Santa Clara, CA, United States). Samples of QWZK were detected in positive ion detection mode, and the spray and capillary voltages were set to 4.0 KV and 35.0 V, respectively. The tube lens voltage was 110 V, and the source temperature was set to 350°C. Nitrogen (purity >99.99%) was used as both the sheath gas (40 arb) and auxiliary gas (20 arb). Then, samples were analyzed in negative ion detection mode, with the spray and capillary voltages set to 3.0 kV and 35.0 V, respectively. The tube lens was set to 110 V, and the source temperature was set to 350°C. Nitrogen (purity >99.99%) was used as both the sheath gas (30 arb) and auxiliary gas (10 arb). Data-dependent acquisition (ddms3) of high-resolution Fourier transform (TF, full scan; resolution, 30,000) and CID fragmentation were used for positive and negative ion data acquisition. The compositions of QWZK were authenticated by referring to the retention time of each chemical component, high-resolution precise molecular weight, and MSn multilevel fragment information detected by LC-MS and combined with the extraction of ion flow map and standard product information and related literature.

### Induction of ALI by LPS

Adult Wistar rats (4–6 weeks, 180–220 g, male) were purchased from the Vital River Laboratories (SYXK 2016-0006). All the animals were housed in an environment with temperature of 23 ± 1°C, relative humidity of 50 ± 1%, and a light/dark cycle of 12/12 h. All animal experimental procedures were conducted in strict accordance with the Guide for the Care and Use of Laboratory Animals and were approved by the Animal Care and Use Committee of Beijing University of Chinese Medicine. After acclimation for 7 days for 7 days, the rats were randomly assigned into 6 groups (*n* = 10/group): the control group was treated by saline only, the LPS group was treated by LPS (from *Escherichia coli* O55:B5, abs47014848, Absin, Shanghai, China) only, the dexamethasone group was treated by dexamethasone and LPS, and the QWZK groups were pretreated with QWZK followed by LPS. QWZK was administrated with 3, 6, and 12 g/kg *via* intragastric (i.g.) administration once per day for 7 consecutive days. LPS was injected intraperitoneally (i.p.) after final injection of medication for 1 h. At 4-h intervals, the left lung was lavaged with cool phosphate-buffered saline (PBS) to collect the bronchoalveolar lavage fluid (BALF); the middle lobe of the right lung was fixed with 4% paraformaldehyde (PFA), and the upper lobe, lower lobe, and accessory lobes were stored at −80°C for protein expression tests.

### WBC Count and Analysis

The BALF was centrifuged at 3,000 rpm for 5 min, at 4°C. The supernatant was discard, and then, the cell pellet was resuspend in PBS. Whereafter, a Sysmex XS-800iBayer ADVIA120 Hematology System was used for cell counting and classification.

### Hematoxylin–Eosin Staining

The tissues fixed with 4% PFA were dehydrated, transparent, and immersed in paraffin. Before staining, the slices (3 μm) were dewaxed and soaked. Then, they were stained with hematoxylin aqueous solution and eosin staining solution, respectively. Finally, they were dehydrated and rendered transparent and sealed with neutral gum. Sections were observed under a microscope and photographed. A 20-fold field was selected for the statistics of alveolar wall area (%). The airway wall area was detected and calculated with Image-Pro Plus software according to references (Moon et al., 2021). We used the lung tissue slices of the control group to calibrate the quantitative parameters, randomly select different areas of the lung, and quantify the alveolar wall and blank area. Alveolar wall ratio (%) = alveolar wall area/total area. Lung injury was scored according to the following criteria (Schingnitz et al., 2010; Moon et al., 2021; Zhao et al., 2021): (1) alveolar congestion, (2) hemorrhage, (3) infiltration or aggregation of neutrophils in airspace or vessel wall, and (4) thickness of the alveolar wall. For each subject, a 5-point scale was applied: 0, minimal (little) damage; 1+, mild damage; 2+, moderate damage; 3+, severe damage; and 4+, maximal damage. The total score of each criteria was used for statistics.

### Western Blot

To investigate the expression of proteins by Western blot analyses, animal tissue samples were lysed in a protein cell lysis buffer (Applygen, Beijing, China). The protein concentration of the samples was determined using a bicinchoninic acid (BCA) protein assay kit (Thermo Fisher Scientific, MA, United States). The samples were boiled for 10 min, and proteins were separated by electrophoresis using a 10% or 12% sodium dodecyl sulfate (SDS)-polyacrylamide gel. After the transfer of protein to a polyvinylidene difluoride membrane (PVDF, Millipore, Bedford, MA, United States), the membrane was incubated in blocking buffer [5% non-fat dairy milk in Tween-20 Tris-buffered saline (TBST)] for 2 h at ambient temperature and probed with various antibodies in a blocking buffer overnight at 4°C. The membrane was washed four times with 0.1% TBST, probed with a secondary antibody in the blocking buffer for 2 h at ambient temperature and then washed again with TBST. The membranes were detected with an enhanced chemiluminescence kit (Amersham Pharmacia Biotech, Piscataway, NJ, United States). The primary antibody included TLR4 (1:1,000, NB100-56566, Novus, CO, United States), IKKα (1:1,000, #11930, CST, Boston, United States), IKKβ (1:1,000, #8943, CST, Boston, United States), p-IKKα/β (1:1,000, #2697, CST, Boston, United States), IκBα (1:1,000, #4814, CST, Boston, United States), p-IκBα (1:1,000, #2859, CST, Boston, United States), NF-κB p65 (1:1,000, #8242, CST, Boston, United States), p-NF-κB p65 (1:1,000, #3033, CST, Boston, United States), NLRP3 (1:1000, ab263899, Abcam, Cambridge, United States), pro-caspase-1 + p10 + p12 (1:1,000, ab179515, Abcam, Cambridge, United States), ASC/TMS1 (1:1,000, NBP1-78977, Novus, Colorado, United States), and β-actin (1:5,000, #8457, CST, Boston, United States). The secondary antibodies include goat antirabbit IgG H&L (HRP, 1:5,000, ab6721, Abcam, Cambridge, United States), goat antimouse IgG H&L (HRP, 1:5,000, ab6789, Abcam, Cambridge, United States).

### ELISA

The contents of IL-6, TNF-α, IFN-γ, MCP-1, IL-1β, and IL-18 were determined by ELISA method. The lung tissues were crushed with balls at 0–4°C and centrifuged at 12,000 rpm for 20 min. Protein concentration was determined by BCA protein assay kit (Thermo Fisher Scientific, MA, United States). The levels of IL-6, TNF-α, IFN-γ, MCP-1, IL-1β, and IL-18 in lung tissues were measured by using commercially procured ELISA assay kits, including IL-6 ELISA kits (Biolegend, San Diego, United States, Item No. 437107), TNF-α ELISA kits (Biolegend, San Diego, United States, Item No. 438207), IFN-γ ELISA kits (Biolegend, San Diego, United States, Item No. 439007), MCP-1/CCL2 ELISA kits (Genie, London, United Kingdom, Item No. RTFI00038), IL-1β ELISA kits (Genie, London, United Kingdom, Item No. RTDL00552), and IL-18 ELISA kits (Genie, London, United Kingdom, Item No. RTDL00548).

### Statistical Analysis

The data and statistical analysis comply with the *British Journal of Pharmacology* on experimental design and analysis in pharmacology (Curtis et al., 2018). All data were presented as means ± standard error of mean (SEM). All rights reserved based on at least three independent experiments and analyzed on GraphPad Prism 8.0 (GraphPad Software, San Diego, CA, United States). Statistical analysis was undertaken for studies where each group rats were at least n = 8. Statistical data conforming to a Gaussian distribution was performed either with one-way analyses of variance (ANOVAs) followed by Fisher’s least significant difference (homogeneity of variances) and Tamhane T2 (heterogeneity of variance) *post-hoc* test using SPSS version 25 for windows (IBM^®^ SPSS^®^ Statistics, Chicago, IL, United States). Mann–Whitney U test was applied to data analysis of abnormal distribution. *p* < 0.05 was considered statistically significant.

## Results

### The Chemical Compositions of QWZK Were Determined

**FIGURE 1:**
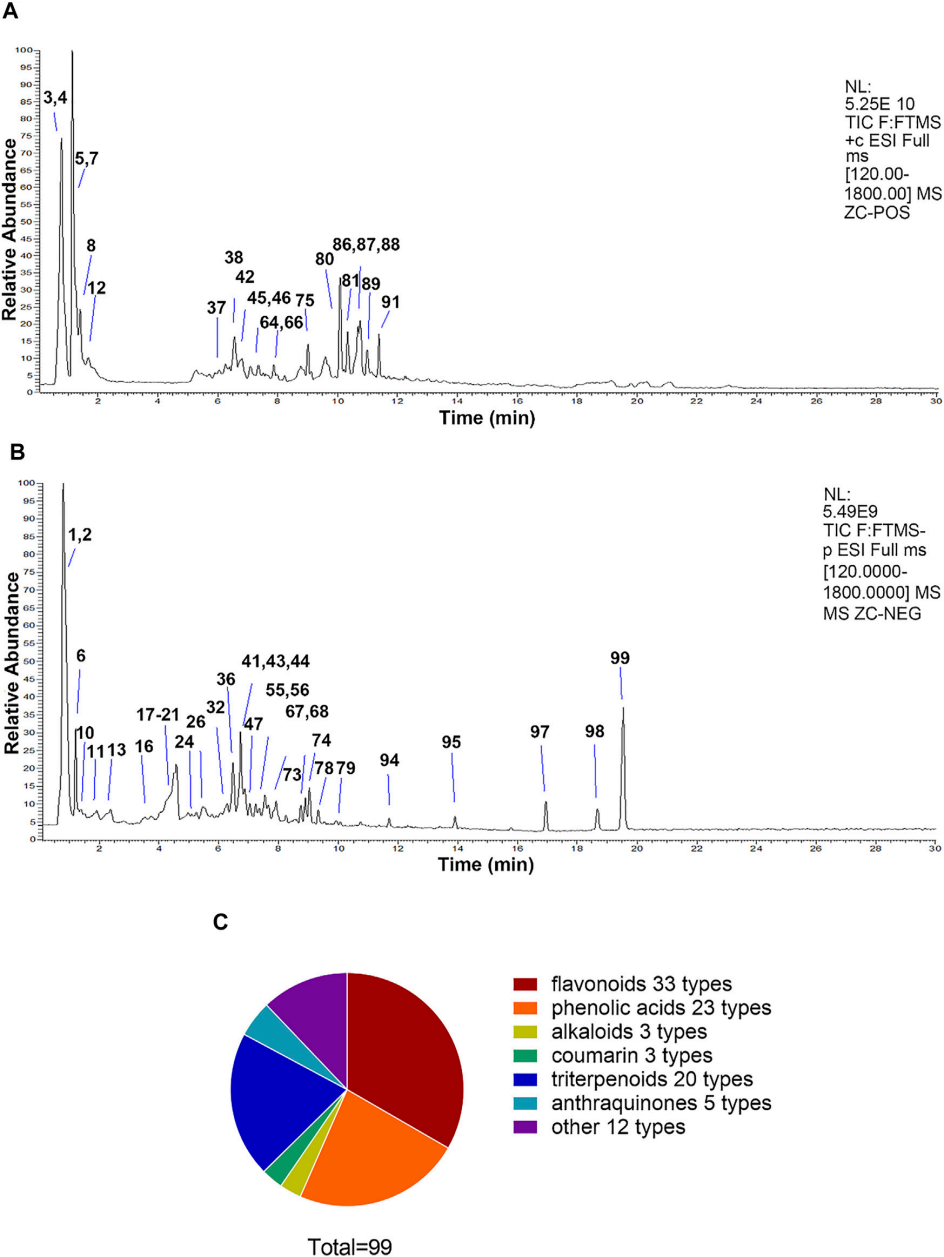
The compositions of QWZK were determined by UHPLC-LTQ-Orbitrap MS. **(A)** Total ion flow diagram of QWZK in positive mode. **(B)** Total ion flow diagram of QWZK in anion mode. **(C)** Number of monomer components in QWZK identified by positive and anion UHPLC-LTQ-Orbitrap MS.

In this study, the chemical compositions of QWZK were analyzed by UHPLC-LTQ-Orbitrap MS. There were 21 compounds identified in the negative spectrum and 78 compounds were identified in the positive spectrum. A total of 99 compounds were identified, including 33 flavonoids, 23 phenolic acids, 3 alkaloids, 3 coumarins, 20 triterpenoids, 5 anthraquinones, and 12 others (Figure 1). The list of identified compounds was shown in the supplementary material (Supplementary Table S1).

### QWZK Recovered the WBC Counts and Classification and Improved Pathological Changes in the Lungs of ALI Rats Induced by LPS

**FIGURE 2:**
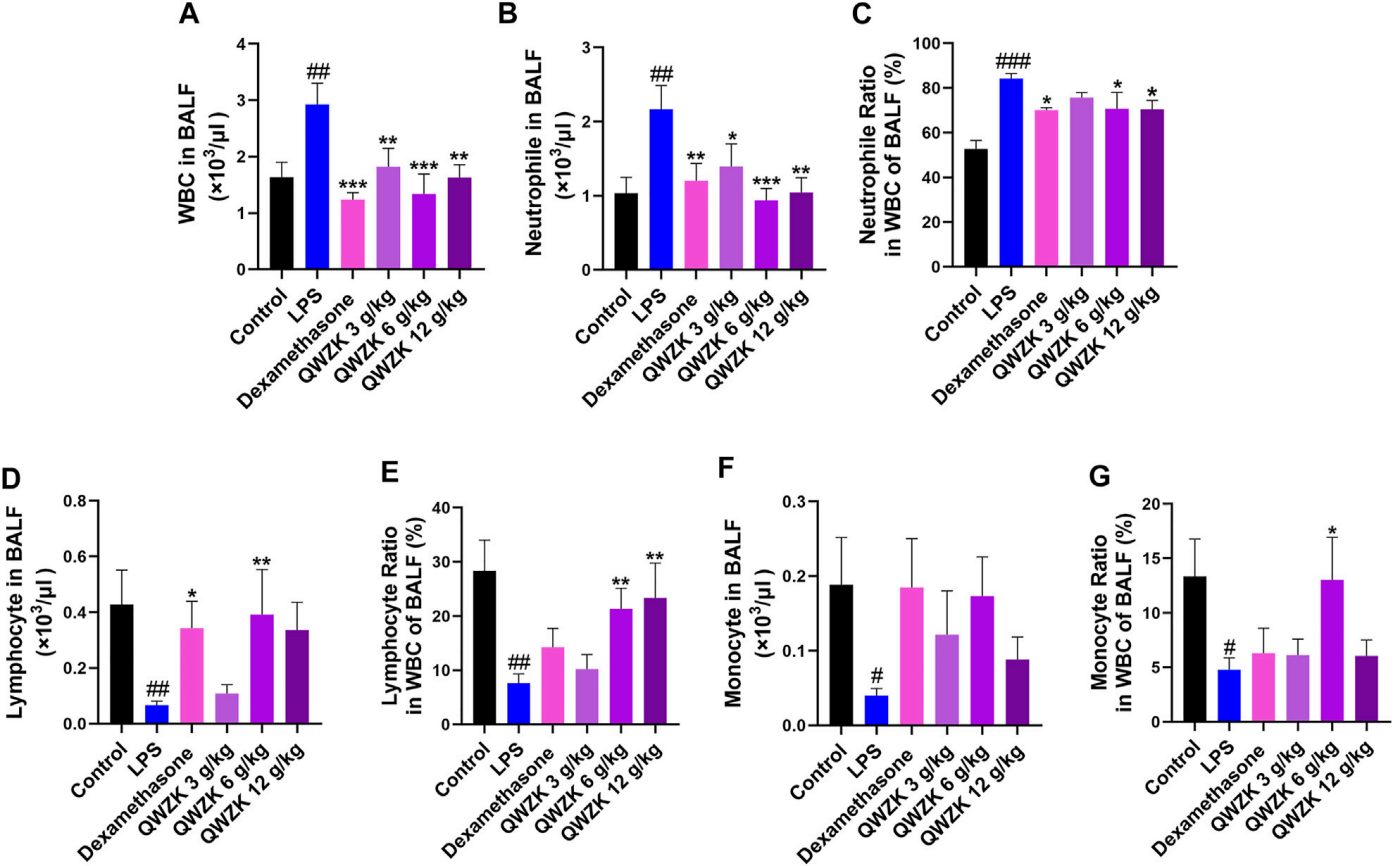
QWZK recovered the WBC counts and classification in ALI rats induced by LPS. **(A)** The number of WBC in BALF was detected by globulimeter. **(B, C)** Globulimeter was used to detect the number and ratio of neutrophils in BALF. **(D, E)** The number and ratio of lymphocyte were detected by a globulimeter in different groups. **(F, G)** The globulimeter was used to detect the number and ratio of monocyte in different groups. Data were presented as the mean ± SEM, *n* ≥ 8. ^#^ *p* < 0.05 *vs*. control group, ^##^ *p* < 0.01 *vs*. control group, ^###^ *p* < 0.001 *vs*. control group. *p < 0.05 *vs*. LPS group, ^**^ *p* < 0.*vs*. LPS group, ^***^ *p* < 0.001 *vs*. LPS group.

The effects of QWZK on ALI were evaluated by the detection of the number and classification of leukocyte in BALF and the histopathology of lung. As shown in Figure 2A, the number (2.92 ± 0.57 × 10^3^ cells/μl) of leukocyte in BALF of the rats in LPS group was significantly increased than that in the control group (1.63 ± 0.26 × 10^3^ cells/μl), and the number of leukocyte in BALF of rats in the dexamethasone group and QWZK 3 g/ kg group, QWZK 6 g/ kg group, and QWZK 12 g/ kg group were 1.24 ± 0.11 × 10^3^ cells/μl, 1.94 ± 0.35 × 10^3^ cells/μl, 1.38 ± 0.41 × 10^3^ cells/μl, and 1.69 ± 0.30 × 10^3^ cells/μl, respectively, which were declined significantly than that in the LPS group (Figure 2A). The number (2.17 ± 0.32 × 10^3^ cells/ μl) and proportion (84.17 ± 2.27%) of neutrophils in BALF of LPS group were enhanced significantly compared with those of control group (1.04 ± 0.21 × 10^3^ cells/μl, 52.77 ± 3.78%). The dexamethasone and QWZK 3 g/ kg, QWZK 6 g/ kg, and QWZK 12 g/kg treatments all decreased the number and proportion of neutrophils in BALF (Figures 2B,C). Moreover, QWZK 6 g/kg decreased the neutrophil number to 0.94 ± 0.16 × 10^3^ cells/μl, as much as that in control group. Furthermore, the number of lymphocyte and monocyte in BALF of rats in the LPS group were 0.03 ± 0.01 × 10^3^ cells/ μl and 0.04 ± 0.01 × 10^3^ cells/ μl, respectively, which declined significantly compared with that in the control group (0.43 ± 0.12 × 10^3^ cells/μl, 0.19 ± 0.06 × 10^3^ cells/ μl), and QWZK treatment increased compared with that of the LPS group (Figures 2D,F) and the changes in proportion (Figures 2C,E,G). All these data suggested that QWZK reduced inflammatory response in LPS-induced ALI rats.

**FIGURE 3:**
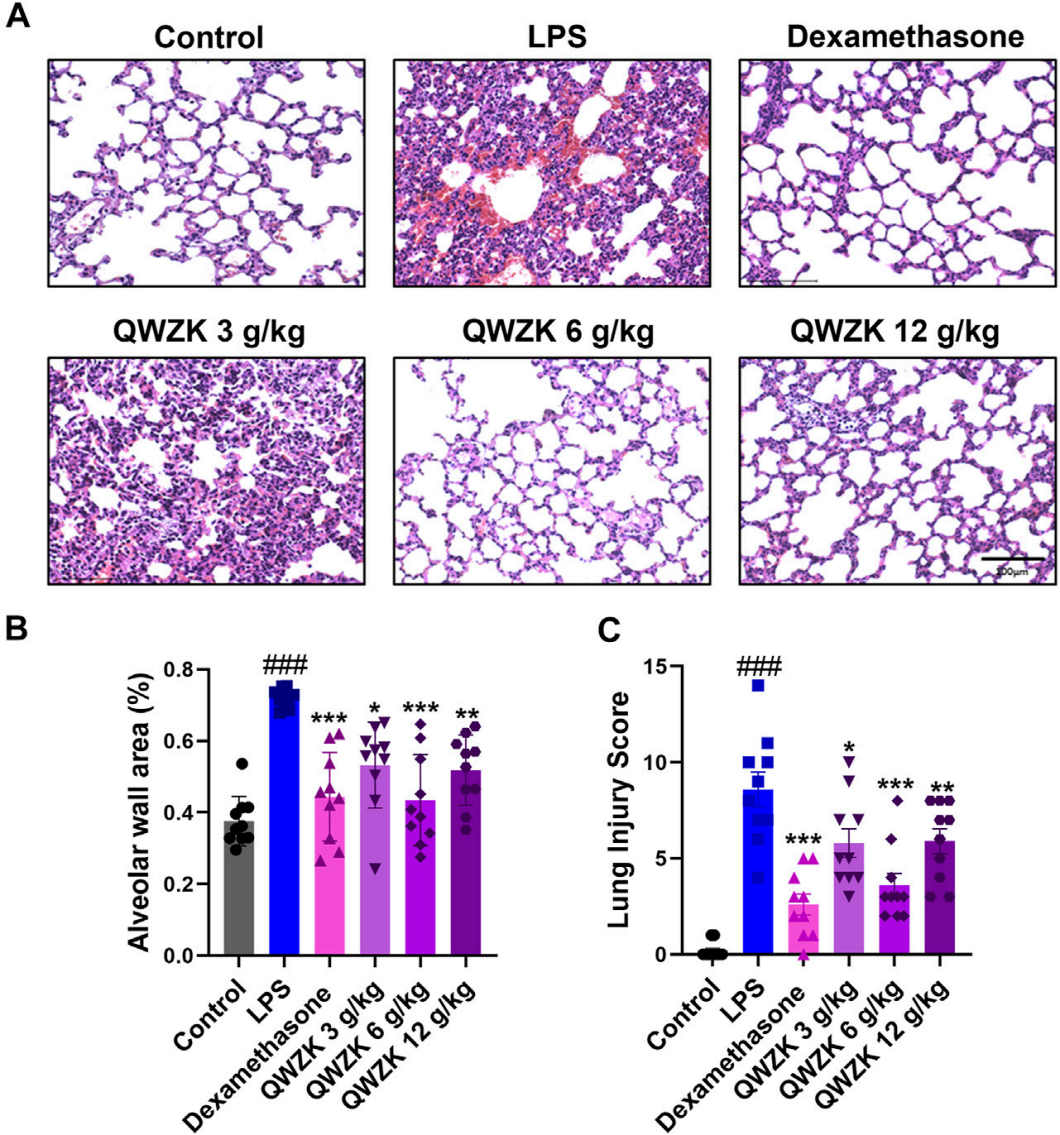
QWZK alleviated the pathological characteristics of lung in ALI rats induced by LPS. **(A)** The images of lung in different groups, which was stained with H&E. Images are representatives of independent experiments. The scale bar in the figures represents a distance of 100 μm. **(B)** Statistical results of alveolar wall percentage in different groups. Data were presented as the mean ± SEM, *n* = 10. **(C)** Statistical results of lung injury score in different groups. Data were presented as the median ± SEM, *n* = 10. ^#^ *p* < 0.05 *vs*. control group, ^##^ *p* < 0.01 *vs*. control group, ^###^ *p* < 0.001 *vs*. control group. *p < 0.05 *vs*. LPS group, **p < 0.01 *vs*. LPS group, ***p < 0.001 *vs*. LPS group.

Besides that, we observed the changes in pulmonary pathology (Figure 3). The alveolar wall areas were performed to evaluate pathological changes in the lung. LPS treatment showed significant thickening of the alveolar wall, thickening of the septum, infiltration of neutrophils in the septum and alveolar cavity, and obvious bleeding in the lung interstitium (Figures 3A,C). The alveolar wall area of rats in the LPS group were almost twofold more than that in control group. In the dexamethasone group, rats showed mild thickening of the alveolar septum and obvious infiltration of neutrophils. The alveolar wall areas were reduced by 32.1% compared with the LPS group. The rats in QWZK groups showed varying degrees of thickening of the alveolar septum and neutrophil infiltration, and no obvious bleeding lungs were seen. The QWZK of dose 6 g/ kg exhibited the most obvious effect on the anesis of alveolar wall thickness and hemorrhage (Figures 3A,B). These data showed that QWZK could ameliorate LPS-induced ALI by regulating the number and classification of WBC in BALF, and the better effect of QWZK was given at 6 g/ kg.

### QWZK Suppressed the Production of Inflammatory Cytokine in the Lungs of ALI Rats Induced by LPS

**FIGURE 4:**
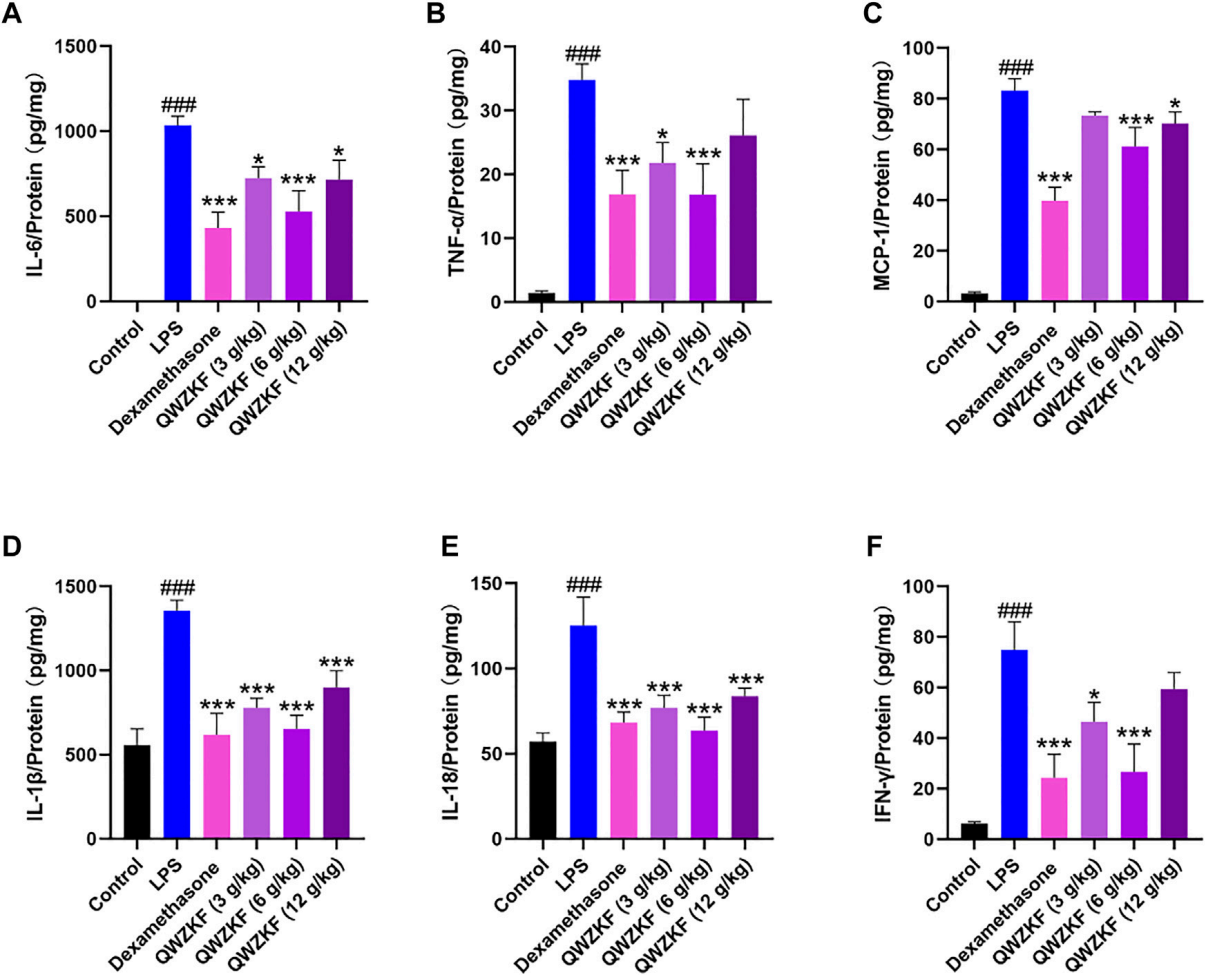
QWZK suppressed inflammatory cytokine levels in ALI rats induced by LPS. The level of **(A)** IL-6, **(B)** TNF-α, **(C)** MCP-1, **(D)** IL-1β, **(E)** IL-18, and **(F)** IFN-γ were detected by ELISA. Data were presented as the mean ± SEM, n ≥ 8. ^#^ *p* < 0.05 vs. control group, ^##^ *p* < 0.01 *vs*. control group, ^###^ *p* < 0.001 *vs*. control group. *p < 0.05 *vs*. LPS group, **p < 0.01 *vs*. LPS group, ***p < 0.001 *vs*. LPS group.

ELISA was performed to evaluate the inflammation-related cytokines, including IL-6, TNF-α, MCP-1, IL-1β, IL-18, and a lymphokine related to immune regulation, IFN-γ, in the total protein of the lung. As shown in Figure 4, the expression of IL-6, TNF-α, IL-1β, and IL-18 of the rats in the LPS group were 1,036.20 ± 53.57 pg/mg, 34.82 ± 2.51 pg/mg, 1,354.90 ± 155.75 pg/mg, and 123.47 ± 42.21 pg/mg, respectively, which were significantly increased compared with those in the control group (2.41 ± 0.68 pg/mg, 1.40 ± 0.34 pg/mg, 556.53 ± 96.13 pg/mg, 57.17 ± 5.03 pg/mg). The expressions of IL-6, TNF-α, IL-1β, and IL-18 in the other groups were lower than those in the LPS group (Figures 4A,B,D,E).

The level of MCP-1 was significantly decreased in the rats after dexamethasone (39.77 ± 13.21 pg/mg), QWZK 3 g/kg (73.39 ± 3.74 pg/mg), QWZK 6 g/kg (61.22 ± 18.07 pg/mg), and QWZK 12 g/kg (70.25 ± 11.02 pg/ mg) administration, respectively, which was in contrast with that of the LPS group (83.17 ± 4.82 pg/ mg). Similarly, LPS induced IFN-γ production, and dexamethasone (24.28 ± 9.38 pg/mg), QWZK 3 g/kg (46.43 ± 7.64 pg/mg), QWZK 6 g/kg (26.63 ± 10.98 pg/mg), and QWZK 12 g/kg (59.34 ± 6.64 pg/mg) treatments could reverse the IFN-γ level to normal (Figure 4F).

Among them, the QWZK 6 g/kg group showed the optimal effect on the downregulation of proinflammatory cytokines, and the inhibition effect on some cytokines, such as TNF-α, IL-18, and IFN-γ, were better than dexamethasone.

### QWZK Inhibited TLR4/NF-κB Pathway in the Lungs of ALI Rats Induced by LPS

**FIGURE 5:**
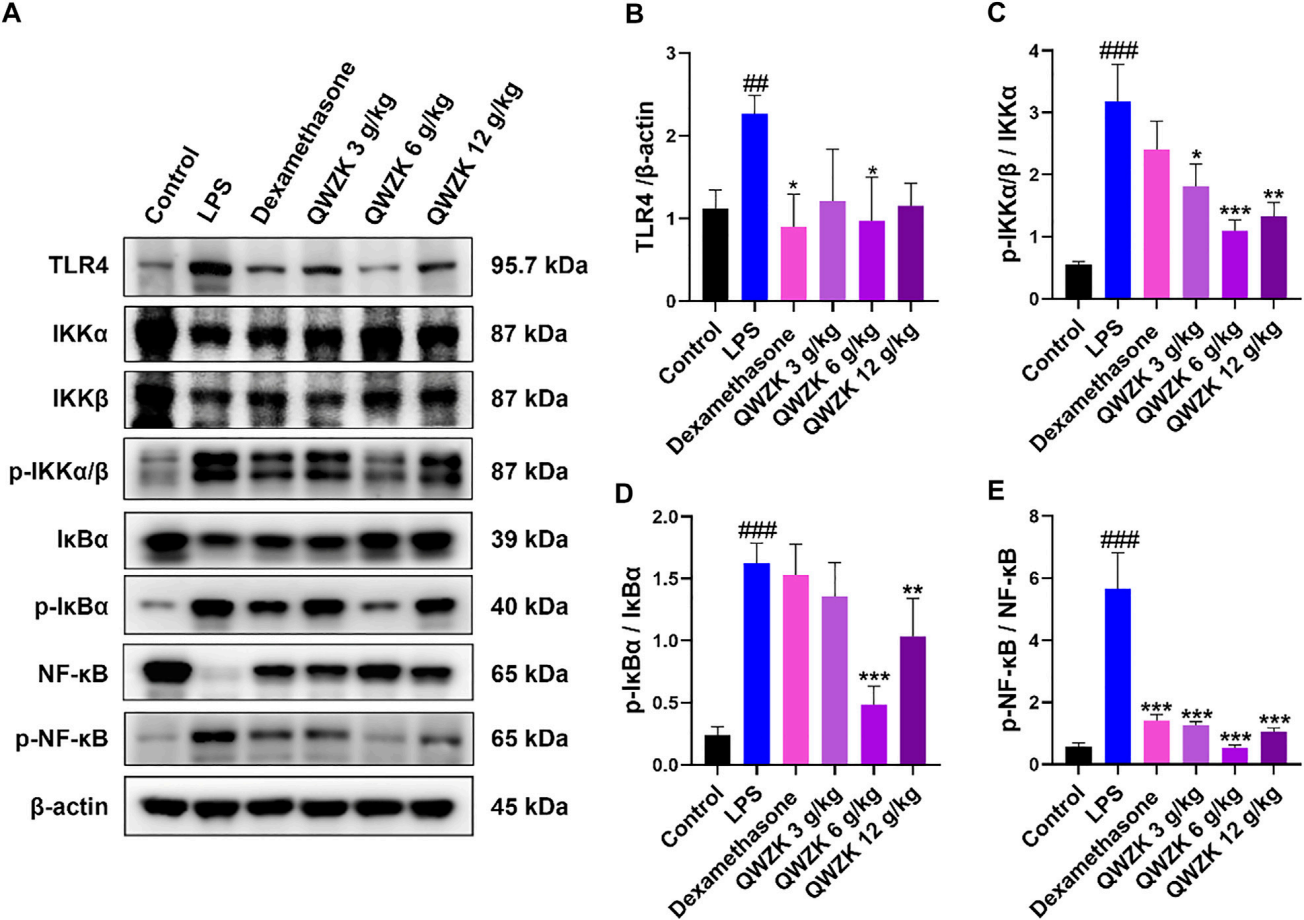
QWZK restrained TLR4/NF-κB pathway in ALI rats induced by LPS. **(A)** The protein expression levels of TLR4, p-IKKα/β, p-IκBα, p- NF-κB, IKKα/β, IκBα, NF-κB, and β-actin were tested by Western blots. **(B–E)** The intensity of proteins bands was quantified. Data were presented as the mean ± SEM, *n* = 8. ^#^ *p* < 0.05 *vs*. control group, ^##^ *p* < 0.01 *vs*. control group, ^###^ *p* < 0.001 *vs*. control group. *p < 0.05 *vs*. LPS group, **p < 0.01 *vs*. LPS group, ***p < 0.001 *vs*. LPS group.

As is well-known, the TLR4/NF-κB signaling pathway is involved in regulating proinflammatory factors (Lawrence and Fong, 2010). Further, we investigated the effect of QWZK on TLR4/NF-κB signaling pathway. As shown in Figure 5, compared with the control group, the expressions of TLR4, p-IKKα/β, p-IκBα, and p-NF-κB were significantly upregulated in the lungs of LPS-induced ALI rats, and IKKα/β, IκBα, and NF-κB expression were significantly downregulated. Compared with the rats in LPS group, TLR4, p-IKKα/β, p-IκBα, and p-NF-κB expression were declined in the dexamethasone group and the QWZK groups. Among them, the expression of TLR4, p-IKKα/β, p-IκBα, and p-NF-κB in QWZK 6 g/kg group was even lower than those in the dexamethasone group and recovered to almost the same level as that in control group. The results indicated that QWZK could inhibit the TLR4/NF-κB signaling pathway.

### QWZK Inhibited NLRP3 Inflammasome Activation in the Lungs of ALI Rats Induced by LPS

**FIGURE 6:**
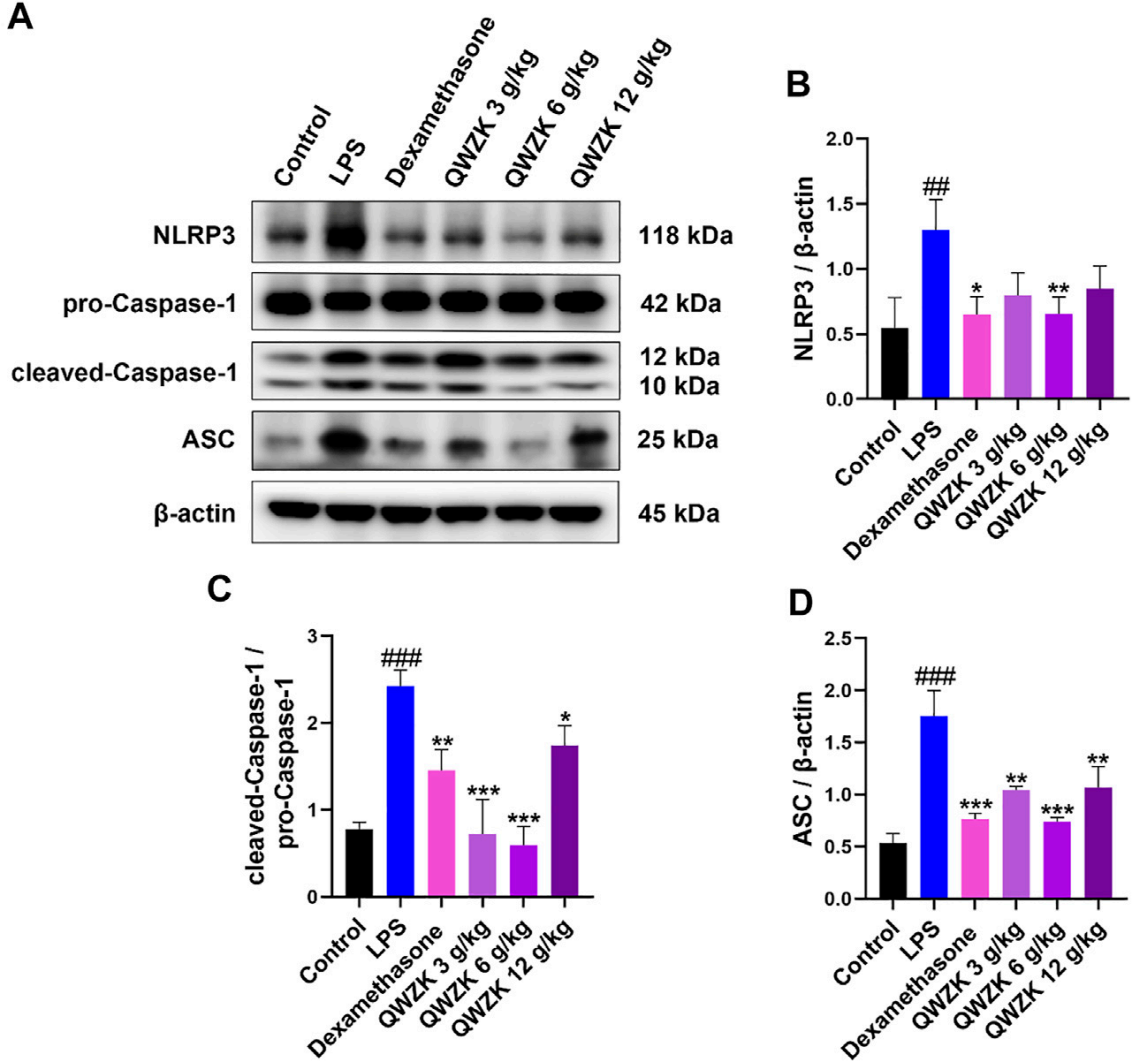
QWZK inhibited NLRP3 inflammasome activation in ALI rats induced by LPS. **(A)** Western blot assay of NLRP3, pro-caspase-1, cleaved caspase-1, and ASC in different groups. **(B–D)** The protein expression was analyzed by gray scale. Data were presented as the mean ± SEM, *n* = 8. ^#^ *p* < 0.05 *vs*. control group, ^##^ *p* < 0.01 vs. control group, ^###^ *p* < 0.001 *vs*. control group. *p < 0.05 *vs*. LPS group, **p < 0.01 *vs*. LPS group, ***p < 0.001 *vs*. LPS group.

Studies demonstrated that inflammasome activation could increase the expression of IL-1β and IL-18 (Seoane et al., 2020). Then, we investigated whether QWZK inhibited IL-1β and IL-18 level *via* activation of NLRP3 inflammasomes. Western blot results showed that the expression of NLRP3, cleaved caspase-1 and ASC increased significantly in the rats of the LPS group compared with those in control group. All of dexamethasone, QWZK 3 g/kg, QWZK 6 g/kg, and QWZK 12 g/kg treatments could downregulate NLRP3, cleaved caspase-1, and ASC expression (Figure 6). Interestingly, the levels of NLRP3 and ASC in the rats treated with QWZK 6 g/ kg were almost the same as those in the rats of the dexamethasone group. QWZK could inhibit the activation of NLRP3 inflammasomes, and QWZK 6 g/kg exhibited better role on inhibition of NLRP3, cleaved caspase-1, and ASC.

## Discussions

We identified 99 compounds in QWZK, including flavonoids, phenolic acids, triterpenoids, anthraquinones, alkaloids, and coumarins. According to literatures reported, some compounds in QWZK play protective roles in the pathogenesis of ALI. For instance, phenolic acid compound–chrysophanol exhibits protective effects of ALI, which were associated with the regulation of the HMGB1/NF-κB pathway *via* HDAC3 (Wang et al., 2020). Coumarin compound, emodin, alleviated LPS-induced pulmonary inflammation in rat lung tissues through inhibiting the mammalian target of rapamycin (mTOR)/hypoxia-inducible factor 1-alpha (HIF-1α)/vascular endothelial growth factor (VEGF) signaling pathway (Li et al., 2020). Triterpenoids, procyanidin B2, significantly suppressed the activation of NLRP3 inflammasome in the lung tissue induced by paraquat in the rat model (Jiang et al., 2018). Platycodin D are protective against LPS-induced ALI by inhibiting NLRP3 and NF-κB signaling pathway (Wu et al., 2021). Flavonoids, chlorogenic acid, markedly decreased activity of inducible nitric oxide synthase (iNOS) in lung tissues, so it prevented nitric oxide (NO) release in response to LPS (Zhang et al., 2010). Rutin is a potential protective agent for ALI *via* inhibition of neutrophil infiltration, expression of vascular cell adhesion molecule 1 (VCAM-1) and iNOS, and NF-κB activation (Yang et al., 2016). Luteolin showed beneficial effects against ALI induced by LPS in mice (Lee et al., 2010). The protective effect of quercetin on ALI involved cAMP-Epac pathway (Wang et al., 2018). Furthermore, octylgallate significantly decreased the iNOS, IL-6, and IL-1β expression and protected alveolar macrophages activated with LPS and on LPS-induced ALI (Haute et al., 2020). Therefore, a variety of components in QWZK could play a protective effect against ALI, and all these evidences supported the hypothesis that QWZK could play protective effects on ALI induced by LPS. In the future, we will quantitatively analyze the components of QWZK and conduct research on the protective effects of the main and higher composition in ALI. On this basis, we lucubrated the protective effect and mechanism of QWZK on ALI.

QWZK is a TCM compound preparation, and the periodic clinical treatment of COVID-19 is 7 days. In this study, the route, dosage and time of QWZK were determined according to the clinical dosage and time, and a single dose of LPS was selected to stimulate rats for too short effective reaction time to construct an ALI model. In order to investigate the protective effect of QWZK on ALI, the treatments of drugs were administrated for 7 days continuously before LPS stimidation. Animal model of ALI induced by LPS in present study exhibited typical characteristics in physiopathological changes as reported (Zhu et al., 2020). Pathological evaluation demonstrated exuberant infiltration and accumulation of WBCs, particularly neutrophils, in both interstitial and alveolar spaces. Analysis of BALF exhibited that the number of WBC and neutrophils were significantly increased, while the number of lymphocytes and monocytes decreased. These results demonstrated that this model of ALI could be used for evaluating protective effects of QWZK on ALI. Furthermore, results of the present study demonstrated the protective effects of QWZK on ALI. QWZK obviously reduced the alveolar wall thickening, hemorrhage, and inflammatory cell infiltration in the interstitial lung tissue and reversed the increase in the WBC and neutrophils and the decrease in the lymphocytes and monocytes in BALF caused by LPS.

Proinflammatory cytokines are key index in severe inflammatory diseases, such as ALI and pneumonia or cytokine storm. These proinflammatory cytokines include interferons (IFNs), tumor necrosis factors (TNFs), interleukins (ILs), and chemokines (Liu et al., 2016). The representative proinflammatory factors IL-6, TNF-α, MCP-1, IL-1β, and IL-18 are highly expressed in inflammatory diseases or cytokine storm. IL-6 and TNF-a are key cytokines in cytokine storm and account for the escalation in aggravation of diseases. MCP-1 is major chemotactic factors for monocytes. IL-1β and IL-18 are secreted by dendritic cells and macrophages, which are activated by NLRP3 inflammasome and cleaved from pro-IL-1β and pro-IL-18. IFN-γ is a lymphokine with strong immunomodulatory properties (Liu et al., 2016; Guo & Thomas, 2017). Once these cytokines increase, they recruit many inflammatory cells, including neutrophils and monocytes. Eventually, inflammatory cells cause an increase in vascular permeability, further aggravating the inflammatory response in the inflammatory disease (McCord et al., 2020). The treatments of cytokine storm can significantly enhance the body to fight against infectious diseases (Rowaiye et al., 2021). In the present study, QWZK significantly downregulated the contents of IL-6, TNF-α, MCP-1, IL-1β, IL-18, and IFN-γ in rat lung of ALI induced by LPS. It suggested that QWZK plays an important role in downregulating the expression of inflammation-related cytokines, in which QWZK 6 g/kg showed the best effect among three test doses on downregulating the expression of IL-6, TNF-α, MCP-1, IL-1β, IL-18, and IFN-γ.

To investigate the underlying mechanisms of protective effects of QWZK on ALI induced by LPS, TLR4/NF-κB signaling pathway was studied. It is well known that LPS can activate the TLR4/NF-κB signaling pathway and initiate the transcription of its downstream inflammatory cytokines IL-6, TNFα, IL-1, and chemokines (Sun, 2017). Our study demonstrated that LPS significantly upregulated the expression of TLR4, p-IKKα/β, p-IκBα, and p-NF-κB but decreased expression of IKKα/β, IκBα, and NF-κB. Other studies reported that LPS induced overexpression of NF-κB or kept constant (Lee et al., 2020; Li et al., 2020; Zhang et al., 2020). To solve this inconsistent question, we tested different dosages and different stimulus times of LPS on NF-κB expression and found that the NF-κB expression was upregulated by LPS on 1 mg/kg at 4 h, 5 mg/kg at both 2 and 4 h, and 10 mg/ kg at 2 h. Meanwhile, the LPS on 2 mg/ kg at 2 h and 10 mg/ kg at 4 h significantly decreased the NF-κB expression (Supplementary Figure S1). These results suggested that the expression of NF-κB induced by LPS showed a trend of dose- and time-dependent manner, but further investigation is needed for their correlation under specific conditions.

The NLRP3 inflammasome is critical for host immune defenses against bacterial, viral, and fungal infections. The activation of NLRP3 inflammasome needs a priming signal. For example, ligands for TLRs or cytokine receptors could activate the transcription factor NF-κB (Kelley et al., 2019). NF-κB could act as the first initiation signal composed of the NLRP3 inflammasome complex and upregulate the expression of NLRP3, caspase-1, pro-IL-1β, and pro-IL-18. Cleaved caspase-1 acts as an activated effector protein, cutting the pro-IL-1β and pro-IL-18 into mature and IL-1β and IL-18, which are secreted to the outside of the cell to mediate inflammation (McVey et al., 2021). Our results demonstrated that QWZK could significantly reduce the expression of NLRP3, cleaved caspase-1, ASC induced by LPS, and the contents of IL-1β and IL-18. QWZK (6 g/kg) could significantly and effectively inhibit the activation of NLRP3 inflammasome and downregulate the level of IL-1β and IL-18. These results suggested that NLRP3 inflammasome is another key mechanism in QWZK protective effects on ALI induced by LPS.

**FIGURE 7:**
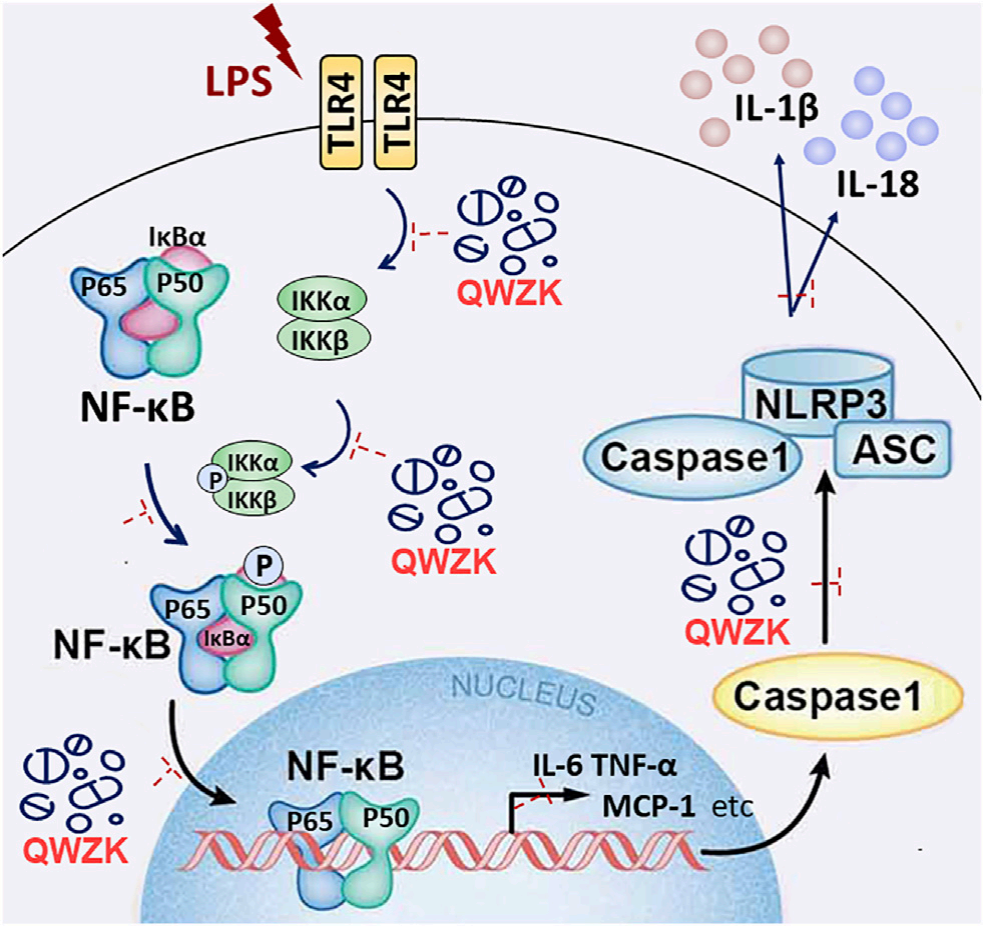
Schematic diagram shows that LPS, as a ligand of TLR4, can activate TLR4/NF-kB pathway and NLRP3 inflammasome and then upregulate the level of IL-6, TNF-α, MCP-1, IFN-γ, IL-1β, and IL-18, promoting lung damage. QWZK could protect LPS-induced ALI *via* downregulating the expression of IL-6, TNF-α, MCP-1, IFN-γ, IL-1β, and IL-18. Its mechanism of action might inhibit TLR4/NF-kB pathway and NLRP3 inflammasome activation.

Generally, our study has verified that LPS could activate the TLR4/NF-kB pathway and NLRP3 inflammasome activation; upregulate the level of some proinflammatory cytokines, chemokines, and lymphokine; and ultimately lead to ALI (Figure 7). QWZK can reduce the WBC and neutrophils in BALF, increase the lymphocytes and monocytes, and ameliorate the pathological process of LPS-induced ALI. The mechanism of QWZK protection against ALI induced by LPS may *via* inhibiting TLR4/NF-kB pathway and NLRP3 inflammasome activation and then downregulated the expression of IL-6, TNF-α, MCP-1, IL-1β, IL-18, and IFN-γ (Figure 7). In present study, the effects of QWZK did not show good dose-effect manners, and similar phenomena were reported in previous studies (Du et al., 2021; Pan et al., 2021; Song et al., 2021; Xie et al., 2022). The possible reason was the components of QWZK are complicated and diversiform, which acted by the mode of multi-component, multi-target and multi-action. The best effects were observed in the middle dose group, which is the clinical equivalent dose. The high dose was twofold of the middle dose. Although its efficacy in anti-inflammation was lower than the middle group, it did not show obvious adverse effects. Considering the complicated compositions of QWZK and the complexity of pathogenesis of ALI, further investigations are needed to elucidate the mechanisms of therapeutic effects of QWZK.

## Conclusion

The chemical compositions of QWZK were first identified. It was demonstrated that QWZK showed protective effects on LPS-induced ALI. The possible underlying mechanisms of QWZK on ALI induced by LPS was *via* inhibiting TLR4/NF-kB signaling pathway and NLRP3 inflammasome activation. Our work suggested that QWZK might be a potential therapeutic candidate for the treatment of ALI and pulmonary inflammation.

## Data Availability

The original contributions presented in the study are included in the article/Supplementary Material. Further inquiries can be directed to the corresponding authors.

## Abbreviations

ALI, acute lung injury; ASC, apoptosis-associated speck-like protein containing CARD; ARDS, acute respiratory distress syndrome; BALF, bronchoalveolar lavage fluid; BSA, body surface area; COPD, chronic obstructive pulmonary disease; CS, corticosteroids; ELISA, enzyme-linked immunosorbent assay; i.g., intragastric administration; i.p., intraperitoneal injection; IL-6, interleukin-6; MCP-1, monocyte chemoattractant protein; Myd88, medullary differentiation protein 88; NF-κB, nuclear factor-κB; NLRP3, NOD-like receptor family pyrin domain containing 3; NO, nitric oxide; PVDF, polyvinylidene difluoride membrane; QWZK, Qingwenzhike Preparation; TBST, Tris-buffered saline Tween-20; TLR4, Toll-like receptor 4; TNF-α, tumor necrosis factor-α; UHPLC-LTQ-Orbitrap MS, linear ion trap/electrostatic field orbital trap tandem high-resolution mass spectrometry.
