# Supplementary material for: Qingwenzhike Prescription Alleviates Acute Lung Injury Induced by LPS via Inhibiting TLR4/NF-kB Pathway and NLRP3 Inflammasome Activation
Source: Front Pharmacol. 2021 Dec 23;12:790072. doi: 10.3389/fphar.2021.790072 (PMC8733650; doi:10.3389/fphar.2021.790072)
Supplement: Supplementary file 1 [file Table1.docx]

**STable.1** UHPLC-LTQ-Orbitrap MS characteristic of major components in tested preparations from QWZK.

| NO. | RT (min) | Measured mass (m/z) | Compound |
| --- | --- | --- | --- |
| 1 | 0.75 | 549.10 [M - H]^-^ | Liquiritin apioside |
| 2 | 0.82 | 191.10 [M - H]^-^ | Isocitric acid |
| 3 | 0.92 | 280.14 [M + H]^+^ | Asparaginyl-Phenylalanine |
| 4 | 1.09 | 175.10 [M + H]^+^ | Arginine |
| 5 | 1.17 | 166.12 [M + H]^+^ | Ephedrine |
| 6 | 1.21 | 191.10 [M - H]^-^ | Citric acid |
| 7 | 1.24 | 180.14 [M + H]^+^ | Methylephedrine |
| 8 | 1.39 | 256.10 [M + H]^+^ | Radicamine A |
| 9 | 1.41 | 169.01 [M - H]^-^ | Gallic acid |
| 10 | 1.62 | 207.05 [M - H]^-^ | 3,4-Dimethoxycinnamic acid |
| 11 | 1.63 | 315.07 [M - H]^-^ | Protocatechuic acid-3-glucoside |
| 12 | 1.66 | 169.0 [M + H]^+^ | Vanillic acid |
| 13 | 2.29 | 353.08 [M - H]^-^ | Neochlorogenic acid |
| 14 | 2.58 | 341.09 [M - H]^-^ | Caffeoyl hexoside |
| 15 | 2.97 | 203.10 [M - H]^-^ | Tryptophan |
| 16 | 3.66 | 341.09 [M - H]^-^ | Caffeoyl-D-glucose |
| 17 | 4.03 | 456.15 [M - H]^-^ | Amygdalin |
| 18 | 4.05 | 353.10 [M - H]^-^ | 4-O-caffeoylquinic acid |
| 19 | 4.27 | 447.09 [M - H]^-^ | Luteolin-7-O-glucoside |
| 20 | 4.45 | 353.08 [M - H]^-^ | Chlorogenic acid |
| 21 | 4.52 | 431.10 [M - H]^-^ | Cosmosiin |
| 22 | 4.94 | 253.1 [M - H]^-^ | Chrysophanol |
| 23 | 4.96 | 179.0 [M - H]^-^ | Caffeic acid |
| 24 | 5.15 | 421.1 [M - H]^-^ | Mangiferin |
| 25 | 5.29 | 577.12 [M + H]^+^ | Procyanidin B |
| 26 | 5.65 | 367.1 [M - H]^-^ | 4-O-feruloylquinic acid |
| 27 | 5.76 | 431.1 [M - H]^-^ | Apigenin-7-O-β-D-glucopyranoside |
| 28 | 5.97 | 497.33 [M - H]^-^ | Poricoic acid A |
| 29 | 6.03 | 609.21 [M - H]^-^ | Hesperidin |
| 30 | 6.05 | 1265.52 [M - H]^-^ | Platycodin C |
| 31 | 6.14 | 1369.64 [M - H]^-^ | Polygalacin D2 |
| 32 | 6.26 | 463.09 [M - H]^-^ | Quercetin hexoside |
| 33 | 6.35 | 193.04 [M - H]^-^ | Ferulic acid |
| 34 | 6.36 | 448.12 [M - H]^-^ | Cyanidin-3-glucoside |
| 35 | 6.40 | 515.12 [M - H]^-^ | 3,5-di-O-caffeoylquinic acid |
| 36 | 6.43 | 843.12 [M - H]^-^ | Platycoside L |
| 37 | 6.44 | 155.04 [M + H]^+^ | Protocatechuate |
| 38 | 6.55 | 611.16 [M + H]^+^ | Isoorientin 7-glucoside |
| 39 | 6.63 | 191.02 [M - H]^-^ | Scopoletin |
| 40 | 6.66 | 1547.68 [M - H]^-^ | Platycoside E |
| 41 | 6.70 | 609.18 [M - H]^-^ | Rutin |
| 42 | 6.71 | 471.20 [M + H]^+^ | Limonin |
| 43 | 6.74 | 325.12 [M - H]^-^ | Coumaric acid glucoside |
| 44 | 6.79 | 1219.40 [M - H]^-^ | Platyconic acid A lactone |
| 45 | 6.97 | 430.33 [M + H]^+^ | Imperialine |
| 46 | 6.99 | 341.09 [M + H]^+^ | Cichoriin |
| 47 | 7.14 | 199.12 [M - H]^-^ | Syringic acid |
| 48 | 7.20 | 339.14 [M - H]^-^ | Esculin |
| 49 | 7.26 | 595.20 [M - H]^-^ | Quercetin-pentoside-hexoside |
| 50 | 7.41 | 1253.59 [M - H]^-^ | Platycoside A |
| 51 | 7.43 | 303.05 [M - H]^-^ | Taxifolin |
| 52 | 7.45 | 681.45 [M - H]^-^ | 3-O-β-D-Glucopyranosylplatycodigenin |
| 53 | 7.46 | 432.35 [M + H]^+^ | Verticine |
| 54 | 7.51 | 463.13 [M - H]^-^ | Isoquercitrin |
| 55 | 7.52 | 1223.58 [M - H]^-^ | Platycodin D |
| 56 | 7.63 | 1237.5 [M - H]^-^ | Platyconic acid A |
| 57 | 7.72 | 463.1 [M - H]^-^ | Hyperoside |
| 58 | 7.73 | 301.0 [M - H]^-^ | Ellagic acid |
| 59 | 7.78 | 515.14 [M - H]- | 4,5-Dicaffeoylquinic acid |
| 60 | 7.79 | 497.11 [M - H]^-^ | 6α-Hydroxypolyporenic acid C |
| 61 | 7.8 | 1265.59 [M - H]^-^ | Platycodin A |
| 62 | 7.87 | 431.07 [M - H]^-^ | Emodin-6-glucoside |
| 63 | 7.88 | 285.00 [M - H]^-^ | Kaempferol |
| 64 | 7.89 | 1281.6 [M + H]+ | 3-O-Acetylplatyconic acid A |
| 65 | 7.91 | 285.04 [M - H]^-^ | Luteolin |
| 66 | 7.92 | 155.04 [M + H]^+^ | 2,4-Dihydroxybenzoic acid |
| 67 | 7.93 | 301.01 [M - H]^-^ | Quercetin |
| 68 | 7.97 | 415.07 [M - H]^-^ | Chrysophanol-1-glucoside |
| 69 | 8.17 | 515.12 [M + H]^+^ | 3,5-Dicaffeoylquinic acid |
| 70 | 8.42 | 301.12 [M - H]^-^ | 5-Methyl kaempferol |
| 71 | 8.58 | 695.52 [M - H]^-^ | 3-O-β-D-glucopyranosylplatycodigenin methyl ester |
| 72 | 8.71 | 299.06 [M - H]^-^ | Tectoridin |
| 73 | 8.79 | 1191.51 [M - H]^-^ | Deapioplatycodin D |
| 74 | 8.82 | 445.10 [M - H]^-^ | Rhein-8-glucoside |
| 75 | 9.00 | 449.09 [M + H]^+^ | Kaempferol-3-O-galactoside |
| 76 | 9.07 | 359.14 [M - H]^-^ | Rosmarinic acid |
| 77 | 9.08 | 491.12 [M - H]^-^ | Caffeoyl-dihydroxyphenyllactoyl-tartaric acid |
| 78 | 9.33 | 269.11 [M - H]^-^ | Aloe emodin |
| 79 | 10.05 | 283.03 [M - H]^-^ | Rhein |
| 80 | 10.09 | 795.19 [M +Na]^+^ | Kaempferol 3-sophorotrioside |
| 81 | 10.12 | 1239.64 [M + H]^+^ | Platycoside H |
| 82 | 10.34 | 403.14 [M + H]^+^ | Nobiletin |
| 83 | 10.68 | 593.13 [M - H]^-^ | Poncirin |
| 84 | 10.69 | 593.13 [M - H] | Luteolin-7-O-rutinoside |
| 85 | 10.70 | 437.19 [M + H]^+^ | Isochinomin |
| 86 | 10.79 | 433.15 [M + H]^+^ | 3,5,6,7,8,3',4'-Heptamethoxy flavone |
| 87 | 10.97 | 373.08 [M + H]^+^ | Tangeretin |
| 88 | 10.98 | 1255.6 [M + H]^+^ | Deapioplatycodin D2 |
| 89 | 11.01 | 395.11 [M + H]^+^ | Aloesone 7-O-glucoside |
| 90 | 11.34 | 1027.48 [M - H]^-^ | Platycodin L |
| 91 | 11.41 | 437.19 [M + H]^+^ | 7-Chloro-3,3',4',5,6,8-hexamethoxyflavone |
| 92 | 11.63 | 269.05 [M - H]^-^ | Apigenin |
| 93 | 11.65 | 269.09 [M - H]^-^ | Emodin |
| 94 | 11.68 | 269.11 [M - H]^-^ | Genistein |
| 95 | 13.89 | 1119.46 [M - H]^-^ | Platycoside O |
| 96 | 15.17 | 283.20 [M - H]^-^ | Emodin-3-methyl ether |
| 97 | 16.91 | 1025.52 [M - H]^-^ | Ageratoside B2 |
| 98 | 18.61 | 827.42 [M - H]^-^ | Platycogenin A |
| 99 | 19.52 | 665.32 [M - H]^-^ | 3-O-β-D-glucopyranosyl polygalacic acid |
