# Supplementary material for: Qingwenzhike Prescription Alleviates Acute Lung Injury Induced by LPS via Inhibiting TLR4/NF-kB Pathway and NLRP3 Inflammasome Activation
Source: Front Pharmacol. 2021 Dec 23;12:790072. doi: 10.3389/fphar.2021.790072 (PMC8733650; doi:10.3389/fphar.2021.790072)
Supplement: Supplementary file 2 [file DataSheet1.docx]

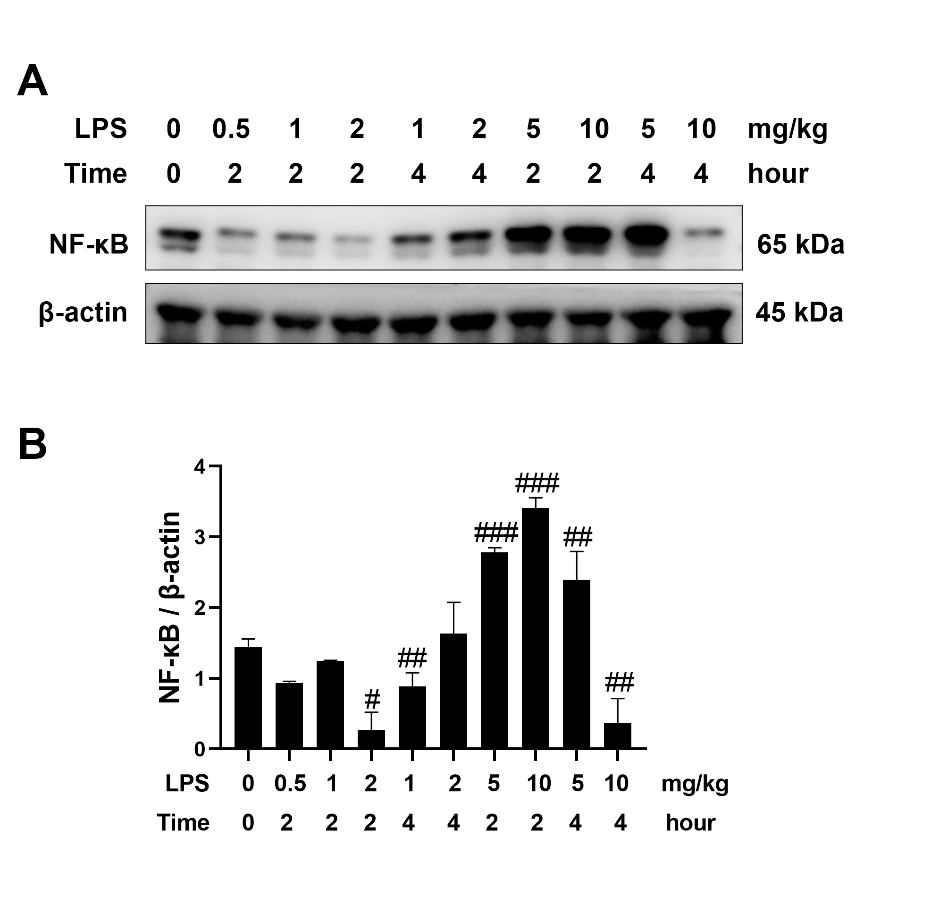


**SFig. 1** LPS regulated the expression of NF-κB in both concentration- dependent manner and time-dependent manner. (**A**) Western blot assay of NF-κB in different groups. (**B**) The protein expression was analyzed by gray scale. Data were presented as the mean ± S.E.M, n=3. ^#^*p* < 0.05 vs. control group, ^##^*p* < 0.01 vs. control group, ^###^*p* < 0.001 vs. control group.

**STable.1** UHPLC-LTQ-Orbitrap MS characteristic of major components in tested preparations from QWZK.

| NO. | RT (min) | Measured mass (m/z) | Compound |
| --- | --- | --- | --- |
| 1 | 0.75 | 549.1 [M - H]^-^ | Liquiritin apioside |
| 2 | 0.82 | 191.1 [M - H]^-^ | Isocitric acid |
| 3 | 0.92 | 280.14 [M + H]^+^ | Asparaginyl-Phenylalanine |
| 4 | 1.09 | 347.1 [M + H]^+^ | Ginkgoic acid |
| 5 | 1.17 | 166.12 [M + H]^+^ | Ephedrine |
| 6 | 1.21 | 191.1 [M - H]^-^ | Citric acid |
| 7 | 1.24 | 180.14 [M + H]^+^ | Methylephedrine |
| 8 | 1.39 | 475.19 [M + H]^+^ | Lucidenic acid B |
| 9 | 1.41 | 169.01 [M - H]^-^ | Gallic acid |
| 10 | 1.62 | 207.05 [M - H]^-^ | 3,4-Dimethoxycinnamic acid |
| 11 | 1.63 | 315.07 [M - H]^-^ | Protocatechuic acid-3-glucoside |
| 12 | 1.66 | 357.19 [M + H]^+^ | Marmesin rutinoside |
| 13 | 2.29 | 353.08 [M - H]^-^ | neochlorogenic acid |
| 14 | 2.58 | 341.09 [M - H]^-^ | Caffeoyl hexoside |
| 15 | 2.97 | 395.16 [M - H]^-^ | Lobetyolin |
| 16 | 3.66 | 341.09 [M - H]^-^ | Caffeoyl-D-glucose |
| 17 | 4.03 | 456.15 [M - H]^-^ | Amygdalin |
| 18 | 4.05 | 353.1 [M - H]^-^ | 4-O-caffeoylquinic acid |
| 19 | 4.27 | 519.1 [M - H]^-^ | Citrusin F |
| 20 | 4.45 | 353.08 [M - H]^-^ | Chlorogenic acid |
| 21 | 4.52 | 502.1 [M - H]^-^ | Gravacridonediol glucoside |
| 22 | 4.94 | 253.1 [M - H]^-^ | Chrysophanol |
| 23 | 4.96 | 179.0 [M - H]^-^ | Caffeic acid |
| 24 | 5.15 | 421.1 [M - H]^-^ | Mangiferin |
| 25 | 5.29 | 366.19 [M + H]^+^ | 4beta-(2-Aminoethylthio)catechin |
| 26 | 5.65 | 367.1 [M - H]^-^ | Curcumin |
| 27 | 5.76 | 431.1 [M - H]^-^ | Apigenin-7-O-β-D-glucopyranoside |
| 28 | 5.97 | 497.33 [M - H]^-^ | poricoic acid A |
| 29 | 6.03 | 577.1 [M - H]^-^ | Procyanidin B2 |
| 30 | 6.05 | 609.2 [M - H]^-^ | Hesperidin |
| 31 | 6.14 | 473.07 [M - H]^-^ | cichoric acid |
| 32 | 6.26 | 463.09 [M - H]^-^ | Quercetin hexoside |
| 33 | 6.35 | 193.0 [M - H]^-^ | ferulic acid |
| 34 | 6.36 | 448.1 [M - H]^-^ | cyanidin-3-glucoside |
| 35 | 6.40 | 515.12 [M - H]^-^ | 3,5-di-O-caffeoylquinic acid |
| 36 | 6.43 | 515.12 [M - H]^-^ | Ganoderic acid B |
| 37 | 6.44 | 579.2 [M + H]^+^ | Narirutin |
| 38 | 6.55 | 611.2 [M + H]^+^ | Isoorientin 7-glucoside |
| 39 | 6.63 | 557.22 [M - H]^-^ | Lobetyolinin |
| 40 | 6.66 | 1547.68 [M - H]^-^ | Platycoside E |
| 41 | 6.70 | 609.2 [M - H]^-^ | Rutin |
| 42 | 6.71 | 432.35 [M + H]^+^ | isoverticine |
| 43 | 6.74 | 609.2 [M - H]^-^ | Neohesperidin |
| 44 | 6.79 | 1219.4 [M - H]^-^ | platyconic acid A lactone |
| 45 | 6.97 | 430.33 [M + H]^+^ | imperialine |
| 46 | 6.99 | 509.89 [M + H]^+^ | 3β-Acetoxy-lanosta-7,9(11),24(31)-trien-21-oic acid |
| 47 | 7.14 | 576.2 [M - H]^-^ | N-acetyldopamine trimers |
| 48 | 7.2 | 521.1 [M - H]^-^ | Iridin |
| 49 | 7.26 | 595.20 [M - H]^-^ | Quercetin-pentoside-hexoside |
| 50 | 7.41 | 1253.59 [M - H]^-^ | Platycoside A |
| 51 | 7.43 | 303.05 [M - H]^-^ | taxifolin |
| 52 | 7.45 | 681.45 [M - H]^-^ | 3-O-β-D-Glucopyranosylplatycodigenin |
| 53 | 7.46 | 432.35 [M + H]^+^ | verticine |
| 54 | 7.51 | 463.1 [M - H]^-^ | Isoquercitrin |
| 55 | 7.52 | 1223.58 [M - H]^-^ | Platycodin D |
| 56 | 7.63 | 1237.5 [M - H]^-^ | platyconic acid A |
| 57 | 7.72 | 463.1 [M - H]^-^ | Hyperoside |
| 58 | 7.73 | 301.0 [M - H]^-^ | Ellagic acid |
| 59 | 7.78 | 414.34 [M + H]^+^ | ebeiedine |
| 60 | 7.79 | 497.1 [M - H]^-^ | 6alpha-Hydroxypolyporenic acid C |
| 61 | 7.8 | 1265.59 [M - H]^-^ | Platycodin A |
| 62 | 7.87 | 431.1 [M - H]^-^ | Emodin-6-glucoside |
| 63 | 7.88 | 285.0 [M - H]^-^ | Kaempferol |
| 64 | 7.89 | 430.33 [M + H]^+^ | verticinone |
| 65 | 7.91 | 285.0 [M - H]^-^ | Luteolin |
| 66 | 7.92 | 414.34 [M + H]^+^ | ebeiedinone |
| 67 | 7.93 | 301.0 [M - H]^-^ | Quercetin |
| 68 | 7.97 | 415.1 [M - H]^-^ | Chrysophanol 1-glucoside |
| 69 | 8.17 | 581.2 [M + H]^+^ | Naringin |
| 70 | 8.42 | 361.2 [M - H]^-^ | Secoisolariciresinol |
| 71 | 8.58 | 695.5 [M - H]^-^ | 3-O-β-D-glucopyranosylplatycodigenin methyl ester |
| 72 | 8.71 | 299.1 [M - H]^-^ | Tectoridin |
| 73 | 8.79 | 455.1 [M - H]^-^ | Physcion-1-O-beta-D-Glucopyranoside |
| 74 | 8.82 | 445.1 [M - H]^-^ | Rhein-8-glucoside |
| 75 | 9.00 | 361.09 [M + H]^+^ | Rosmarinic acid D |
| 76 | 9.07 | 359.1 [M - H]^-^ | rosmarinic acid |
| 77 | 9.08 | 491.12 [M - H]^-^ | Caffeoyl-dihydroxyphenyllactoyl- tartaric acid |
| 78 | 9.33 | 269.1 [M - H]^-^ | Aloe emodin |
| 79 | 10.05 | 283.0 [M - H]^-^ | Rhein |
| 80 | 10.09 | 795.19 [M +Na]^+^ | Kaempferol 3-sophorotrioside |
| 81 | 10.12 | 387.11 [M + H]^+^ | N-acetyldopamine dimer A |
| 82 | 10.34 | 403.14 [M + H]^+^ | Nobiletin |
| 83 | 10.68 | 593.1 [M - H]^-^ | Poncirin |
| 84 | 10.69 | 593.13 [M - H] | Luteolin-7-O-rutinoside |
| 85 | 10.70 | 437.19 [M + H]^+^ | Isochinomin |
| 86 | 10.79 | 433.15 [M + H]^+^ | 3,5,6,7,8,3',4'-Heptamethoxy flavone |
| 87 | 10.97 | 373.1 [M + H]^+^ | Tangeretin |
| 88 | 10.98 | 373.1 [M + H]^+^ | Sinensetin |
| 89 | 11.01 | 395.11 [M + H]^+^ | Aloesone 7-O-glucoside |
| 90 | 11.34 | 383.08 [M - H]^-^ | 5α-cholesta-8,24-dien-3-ol |
| 91 | 11.41 | 437.19 [M + H]^+^ | 7-Chloro-3,3',4',5,6,8-hexamethoxyflavone |
| 92 | 11.63 | 269.05 [M - H]^-^ | Apigenin |
| 93 | 11.65 | 269.1 [M - H]^-^ | Emodin |
| 94 | 11.68 | 269.1 [M - H]^-^ | Genistein |
| 95 | 13.89 | 233.16 [M - H]^-^ | lobetyol |
| 96 | 15.17 | 283.2 [M - H]^-^ | Emodin-3-methyl ether |
| 97 | 16.91 | 351.2 [M - H]^-^ | Coriandrone D |
| 98 | 18.61 | 339.2 [M - H]^-^ | 2',7-Dihydroxy-4'-methoxy-8-prenylflavan |
| 99 | 19.52 | 379.2 [M - H]^-^ | 3-O-Methylglycyrol |
